# Efficacy and liver function preservation with donafenib combined with transarterial chemoembolization in unresectable hepatocellular carcinoma: An ALBI grade analysis

**DOI:** 10.3389/fonc.2025.1612477

**Published:** 2025-08-29

**Authors:** Jinpeng Li, Yan Li, Yuanming Li, Jiasheng Du, Lujun Zhao, Jingtao Zhong

**Affiliations:** ^1^ Department of Radiation Oncology, Key Laboratory of Cancer Prevention and Therapy, Tianjin Medical University Cancer Institute & Hospital, National Clinical Research Center for Cancer, Tianjin’s Clinical Research Center for Cancer, Tianjin, China; ^2^ Department of Interventional Therapy I, Shandong Cancer Hospital and Institute, Shandong First Medical University and Shandong Academy of Medical Sciences, Jinan, Shandong, China; ^3^ Department of Radiology, Shanghe County People’s Hospital, Jinan, China; ^4^ Interventional Vascular Department, Laizhou Hospital of Traditional Chinese Medicine, Laizhou, Shandong, China; ^5^ Department of Oncology, Linqing People’s Hospital, Liaocheng, China; ^6^ Department of Hepatobiliary Surgery, Shandong Cancer Hospital and Institute, Shandong First Medical University and Shandong Academy of Medical Sciences, Jinan, Shandong, China

**Keywords:** hepatocellular carcinoma, ALBI grade, donafenib, transarterial chemoembolization, hepatic function preservation

## Abstract

**Purpose:**

This study evaluated the hepatoprotective effects and safety profile of donafenib combined with transarterial chemoembolization (TACE) in unresectable hepatocellular carcinoma (HCC) through analysis of albumin-bilirubin (ALBI) score modifications.

**Patients and methods:**

We enrolled 36 patients with unresectable HCC receiving donafenib+TACE, with or without immune checkpoint inhibitors. ALBI grades were assessed at baseline and throughout treatment, with statistical analyses examining associations between ALBI grade changes, tumor response, and survival outcomes.

**Results:**

Our prospective analysis demonstrated significant antitumor efficacy with favorable hepatic safety. The regimen achieved objective response and disease control rates of 69.4% and 91.7%, respectively, with median progression-free survival of 10.7 months and median time to response of 1.4 months, indicating rapid therapeutic onset. Hepatic function remained stable throughout treatment, with consistent ALBI grades from baseline to progression/final follow-up (−2.41 ± 0.41 *vs*. −2.45 ± 0.52, P=0.67). Among patients, 58.3% maintained stable hepatic function, while 25.0% improved from ALBI grade 2 to 1. Hepatic function deterioration occurred less frequently in responders than nonresponders (12.0% *vs*. 27.3%). ALBI grade modification emerged as a potential predictive biomarker (AUC=0.665). Patients with ALBI grade below the −0.18 threshold achieved superior clinical outcomes, including higher response rates (84.6% *vs*. 56.5%) and longer progression-free survival (not reached *vs*. 5.63 months, P=0.017). These findings establish hepatic function preservation as both a safety indicator and efficacy predictor for donafenib+TACE therapy.

**Conclusion:**

The combination of donafenib and TACE demonstrates favorable hepatic function preservation while maintaining therapeutic efficacy. ALBI grade improvement is correlated with enhanced treatment response and survival outcomes, indicating potential synergistic benefits for tumor control and hepatic function preservation.

## Introduction

Hepatocellular carcinoma (HCC) constitutes 75%–85% of primary liver cancer cases and represents a significant global health burden (1). The therapeutic landscape for unresectable HCC has substantially evolved, with transarterial chemoembolization (TACE) established as the standard treatment for intermediate-stage disease, whereas targeted therapies and immunotherapy have emerged as effective systemic approaches (2).

The preservation of hepatic function during antitumor therapy remains a critical challenge in HCC management. Clinical data indicate that 30%–40% of patients undergoing TACE treatment experience liver function deterioration, often necessitating premature treatment discontinuation (3). Bruix et al. reported that maintaining liver function during systemic therapy is crucial, as hepatic impairment considerably affects treatment tolerance and survival outcomes (4, 5). Moreover, the complex association between tumor progression and liver function deterioration poses a unique challenge in HCC treatment, highlighting the need for therapeutic strategies that can effectively target tumors while preserving hepatic function.

In recent years, targeted therapy, immunotherapy, and other innovative methods have provided new treatment options for patients with advanced liver cancer, and these methods have become the standard first-line treatment for patients with advanced HCC (6–9). Donafenib, an oral small-molecule multitarget tyrosine kinase inhibitor (TKI), is an improved form of sorafenib, with significantly enhanced molecular stability and improved pharmacokinetics (10). It is also the only single-agent targeted drug superior to sorafenib in first-line head-to-head studies on advanced HCC, characterized by enhanced molecular stability and improved hepatic safety (11). The phase III ZGDH3 trial showed superior overall survival (OS) with donafenib compared with sorafenib (12.1 *vs*. 10.3 months, hazard ratio = 0.831, *P* = 0.0245) and a lower incidence of drug-related adverse events (AEs, 85.3% *vs*. 95.5%) (12). In addition, the REFLECT study established the importance of baseline liver function in treatment outcomes, with the Child–Pugh A status correlating with superior survival rates (7). These findings indicate that combination strategies incorporating newer targeted agents may optimize treatment outcomes while maintaining liver function.

The current study investigated the hepatoprotective effects of donafenib + TACE in unresectable HCC. It also assessed the hypothesis that albumin–bilirubin (ALBI) score modifications may serve as safety indicators and outcome predictors, potentially contributing to optimized treatment strategies for this challenging disease (13). Understanding these parameters may contribute to the development of evidence-based protocols for this therapeutically challenging disease.

## Materials and methods

### Patient selection

This prospective, single-arm, single-center phase II clinical trial was initiated by the researcher. The study participants were recruited from patients at the Affiliated Tumor Hospital of Shandong First Medical University. This study was conducted in accordance with the Declaration of Helsinki. The study was approved by the Ethics Committee of Shandong Cancer Hospital and Institute, and all patients provided informed consent.

The inclusion criteria were (1) patients with liver cancer who strictly met the clinical diagnostic criteria for primary liver cancer according to the Guidelines for the Diagnosis and Treatment of Hepatocellular Carcinoma (2019 Edition) (14) or were diagnosed by histopathology or cytology, (2) patients who received TACE + donafenib or TACE + donafenib with immune checkpoint inhibitors as the first-line treatment, (3) patients aged 18–75 years with a survival time >3 months, (4) patients with an Eastern Cooperative Oncology Group performance status (ECOG PS) of 0–1 before TACE and Child–Pugh grade A or B, and (5) patients with at least one measurable lesion.

The exclusion criteria were (1) patients who had previously undergone systemic antitumor therapy; (2) those with diffuse liver cancer; (3) those with intractable hepatic encephalopathy, refractory ascites, or hepatorenal syndrome; (4) those with previous tumor history; and (5) those with incomplete data.

### Treatment protocol

According to the discretion of the investigator, TACE was administered conventionally or using drug-eluting beads. The administration of multiple TACE treatments depended on evidence of viable tumors or intrapathological recurrence, as observed by contrast-enhanced computed tomography (CT) or magnetic resonance imaging (MRI).

An immune checkpoint inhibitor (carrelizumab or tislelizumab: 200 mg, once every 3 weeks, 1 cycle) was administered within 1–2 weeks following TACE. Donafenib was orally administered following a standardized protocol across all patients, with consistent initiation 3 days after TACE completion in all 36 patients. This standardized 3-day interval was established to allow for initial post-TACE recovery while minimizing treatment delay. All patients began treatment with donafenib at the standard dose of 200 mg twice daily (400 mg total daily dose). Dose adjustments were implemented based on individual patient tolerance: patients with tolerable donafenib-related adverse reactions continued the standard dose of 200 mg twice daily, while those experiencing intolerable but manageable side effects had their dose reduced to 200 mg once daily (50% dose reduction). In cases of severe adverse events or intolerable toxicities, drug administration was temporarily suspended with potential for re-initiation at a reduced dose, or permanently discontinued based on clinical assessment. All dose modification decisions were made through multidisciplinary team discussion involving the treating oncologist, interventional radiologist, and clinical pharmacist.

### Evaluation of treatment response and follow-up

All patients underwent systematic follow-up evaluations at 4–6 week intervals until reaching either the OS endpoint or the final follow-up date (December 31, 2023). The follow-up protocol included comprehensive clinical assessments before and after each treatment cycle, including routine blood analysis, liver and kidney function tests, ALBI grade, tumor markers, electrocardiogram, urinalysis, and blood biochemistry panel. ALBI grades were calculated using the original Johnson et al (13) equation: ALBI grade = (log_10_bilirubin x 0.66) + (albumin x -0.085), where bilirubin is measured inμmol/L and albumin in g/L. ALBI grades were categorized as Grade 1 (≤-2.60), Grade 2 (more than -2.60 to≤-1.39), and Grade 3 (more than -1.39), with lower scores indicating better liver function. ALBI grade change (ALBISC) was defined as the difference between baseline and final follow-up/progression ALBI grades. ALBI grades were assessed at specific timepoints: (1) baseline within 7 days before first TACE, (2) 7–10 days after each TACE session, (3) 4–6 weeks after starting donafenib, (4) every 4–6 weeks during active treatment, and (5) at disease progression or last follow-up.

Imaging surveillance consisted of contrast-enhanced abdominal CT or MRI and chest CT scans performed every 4–8 weeks. Patients with viral hepatitis received standard antiviral medication throughout the follow-up period, with detailed records maintained for all AEs through outpatient visits, inpatient assessments, and telephone consultations as needed.

The treatment response was independently evaluated by two experienced radiologists (>10 years of diagnostic experience) using the modified Response Evaluation Criteria in Solid Tumors (mRECIST) (15). The objective response rate (ORR) was calculated as the sum of the complete response (CR) and partial response (PR), whereas the disease control rate (DCR) was defined as the aggregate of CR, PR, and stable disease (SD). OS was measured from treatment initiation to either death from any cause or the last follow-up date, whereas progression-free survival (PFS) was defined as the duration from treatment initiation to disease progression, death from any cause, or the final follow-up assessment. To evaluate ALBISC as a potential predictor of treatment response, a receiver operating characteristic (ROC) curve analysis was performed. The analysis incorporated two key variables: ALBISC, defined as the change in the ALBI grade from baseline to disease progression, and treatment response, which was categorized by the ORR. ROC analysis was used to establish an optimal ALBISC threshold value, and the corresponding sensitivity and specificity values were determined. Furthermore, treatment-related AEs were systematically documented and graded according to the National Cancer Institute Common Terminology Criteria for Adverse Events version 5.0 (16), with treatment modifications implemented based on AE severity or the development of intolerable toxicities.

### Statistical methods

SPSS 26.0 (IBM Corporation, Somers, NY) and R 3.4.1 (http://www.R-project.org) were used to perform statistical analyses and calculate the optimal cutoff value for the ALBI grade. Continuous variables were presented as mean ± standard deviation or median with interquartile range, whereas categorical variables were expressed as frequencies and percentages. Between-group comparisons were performed using Student’s *t*-test for continuous variables and the chi-squared test or Fisher’s exact test for categorical variables. The Kaplan–Meier method was used to plot survival curves, and log-rank tests were used to compare differences in survival between groups. The prognostic risk factors for OS and PFS were analyzed via Cox regression. *P*-values <0.05 were considered statistically significant.

## Results

### Patient characteristics

**Figure 1:**
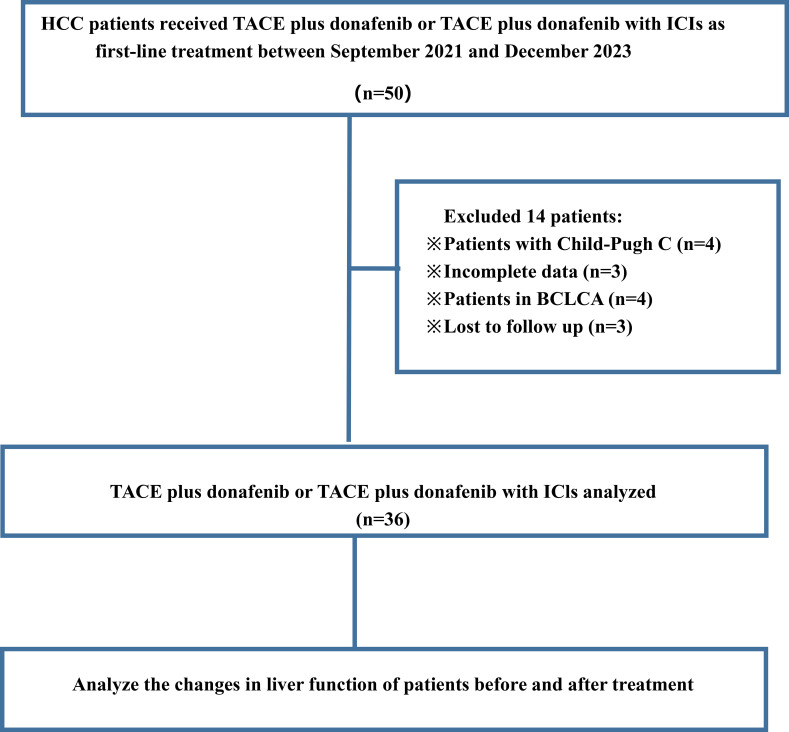
Flowchart of patient selection process showing HCC patients receiving TACE plus donafenib or with ICIs between September 2021 and December 2023.

**Figure 2:**
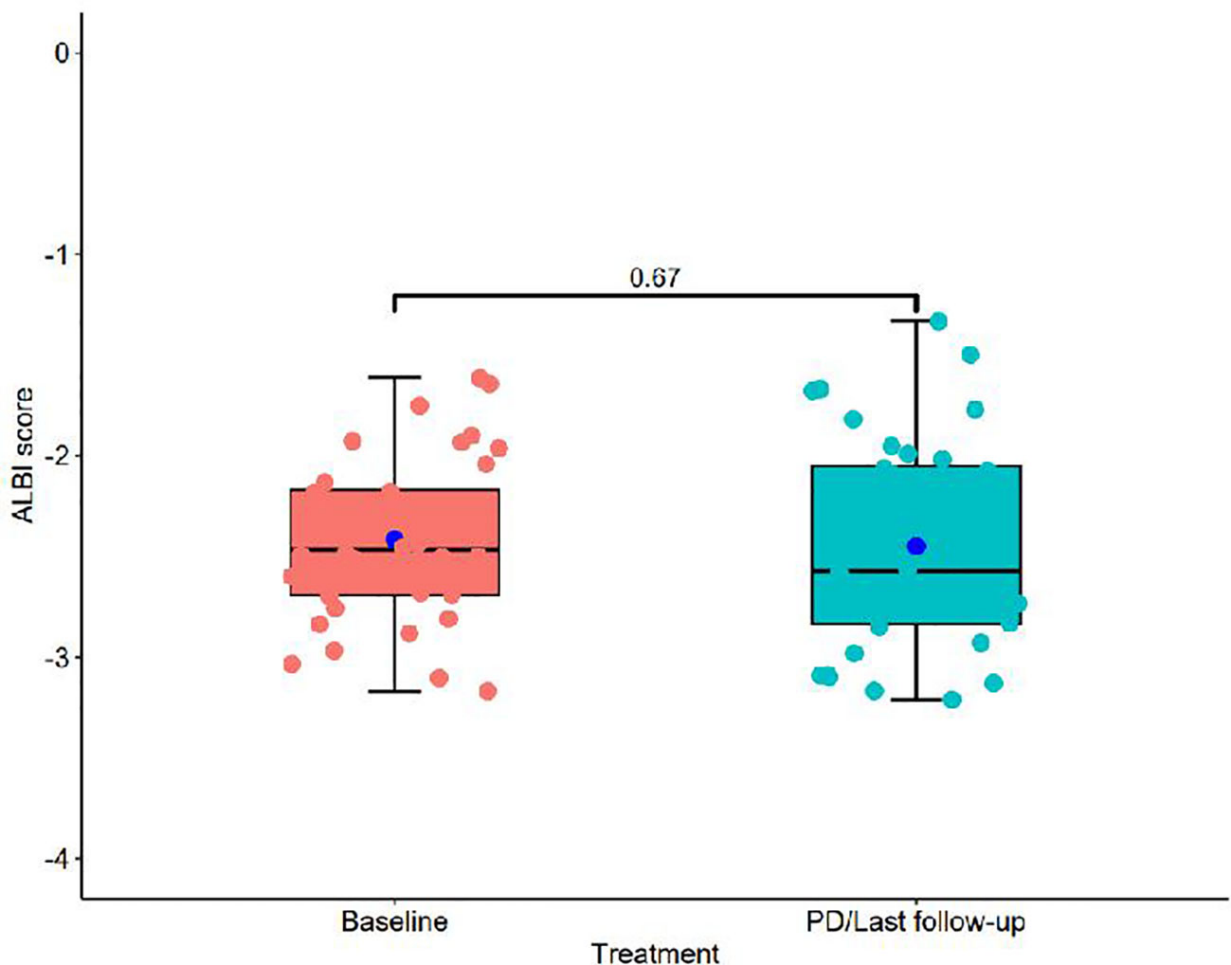
Box plot comparing ALBI scores between baseline and PD/Last follow-up treatments.

**Figure 1** presents a flowchart of the participant selection process. From September 2021 to December 2023, 50 patients received donafenib + TACE with or without an immune checkpoint inhibitor. Screening according to the inclusion criteria resulted in the exclusion of 14 patients, including 3 because of incomplete data and 3 because of no follow-up data. The remaining 36 patients were included in the final analysis (TACE + donafenib, n = 26; TACE + donafenib + immune checkpoint inhibitors, n = 10). Appropriate PD-1 drugs were selected according to the patient’s medical insurance and condition. Of the 36 patients, 33 were men (91.7%), and their median age was 62.0 years. There were 17 (47.2%) patients with an ECOG PS of 1. The complications included hepatitis B virus infection (n = 33, 91.7%) and portal vein tumor thrombus (n = 11, 30.6%). A total of 18 (50.0%) patients had baseline alpha-fetoprotein levels ≥400 ng/mL. Furthermore, 11 patients (30.6%) had a baseline ALBI grade of 1, and 23 (63.9%) had a grade of 2. The institutional distribution is presented in **Table 1**. In this study, the baseline ALBI grade of the patients was −2.41 ± 0.41. At the final observation, the ALBI grade was −2.45 ± 0.52 (*P* = 0.67), indicating no significant difference (**Figure 2**). In addition, we evaluated the relationship between changes in ALBI grade and treatment response as well as survival outcomes. Of the patients, 58.3% maintained stable liver function, and 25% exhibited improvement in their ALBI grades from 2 to 1. These findings clearly demonstrate the superiority of donafenib in improving liver function and its excellent safety profile. The institutional distribution is presented in **Table 2**.

**Table 1:** Baseline characteristics of patients.

Characteristic	All patients (N = 36)
Age (years), median (IQR)	62 (52–69)
Sex	
Male	33 (91.7%)
Female	3 (8.3%)
ECOG PS	
0	19 (52.8%)
1	17 (47.2%)
China liver cancer staging(CNLC)	
Ib	9 (25.0%)
IIa	3 (8.3%)
IIb	13 (36.1%)
IIIa	7 (19.4%)
IIIb	4 (11.1)
HBsAg positive	33 (91.7%)
Child–Pugh class	
A	32 (88.9%)
B	4 (11.1%)
AFP (ng/mL)	
≥400	18 (50.0%)
<400	13 (36.1%)
NA	5 (13.9%)
ALBI score	
1	11 (30.6%)
2	23 (63.9%)
NA	2 (5.5%)
Vascular invasion	
Yes	11 (30.6%)
No	25 (69.4%)
Maximum tumor diameter (cm), median (IQR)	9.1 (5.2–12.4)
Extrahepatic metastasis, n (%)	
Yes	4 (11.1%)
No	32 (88.95)
Type of combination therapy	
TACE+D	26 (72.2%)
TACE+D+I	10 (27.8%)

Unless indicated otherwise, values are presented as n (%).

IQR, interquartile range; ECoG PS, Eastern Cooperative Oncology Group performance status; HBsAg, hepatitis B surface antigen; AFP, alpha-fetoprotein; ALBI, albumin-bilirubin; TACE+D, transarterial chemoembolization plus donafenib; TACE+D+I, transarterial chemoembolization plus donafenib with immune checkpoint inhibitors.

**Table 2:** Tumor response according to mRECIST.

The changes in ALBI score	N	PD patiens	CR+PR	SD+PD
no change	21(58.3%)	11(52.4%)	14(56.0%)	7(63.6%)
2 score change to 1 score	9(25.0%)	2(22.2%)	8(32.0%)	1(9.1%)
1 score change to 2 score	5(13.9%)	3(60.0%)	3(12.0%)	2(18.2%)
2 score change to 3 score	1(2.8%)	1(100%)	0(0)	1(9.1%)

Unless indicated otherwise, values are presented as n (%).

ALBI, albumin–bilirubin; CR, complete response; PR, partial response; SD, stable disease; PD, progressive disease.

#### Effectiveness analysis

**Figure 3:**
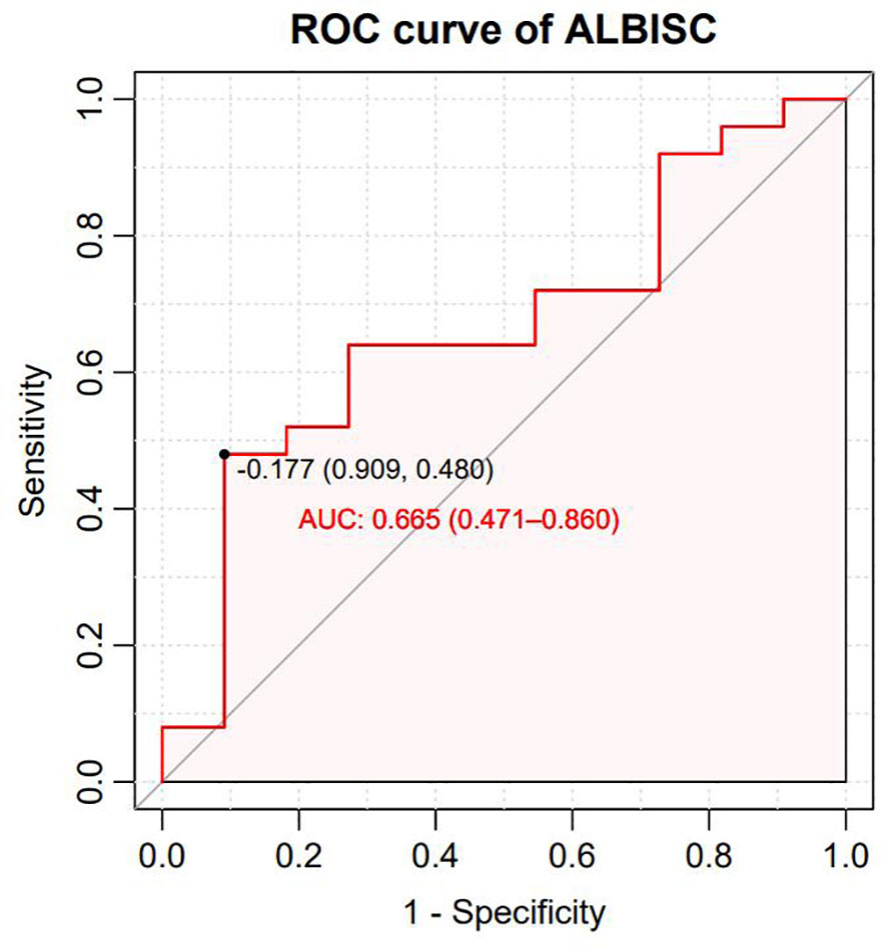
ROC curve for ALBISC with sensitivity plotted against 1-specificity.

**Figure 4:**
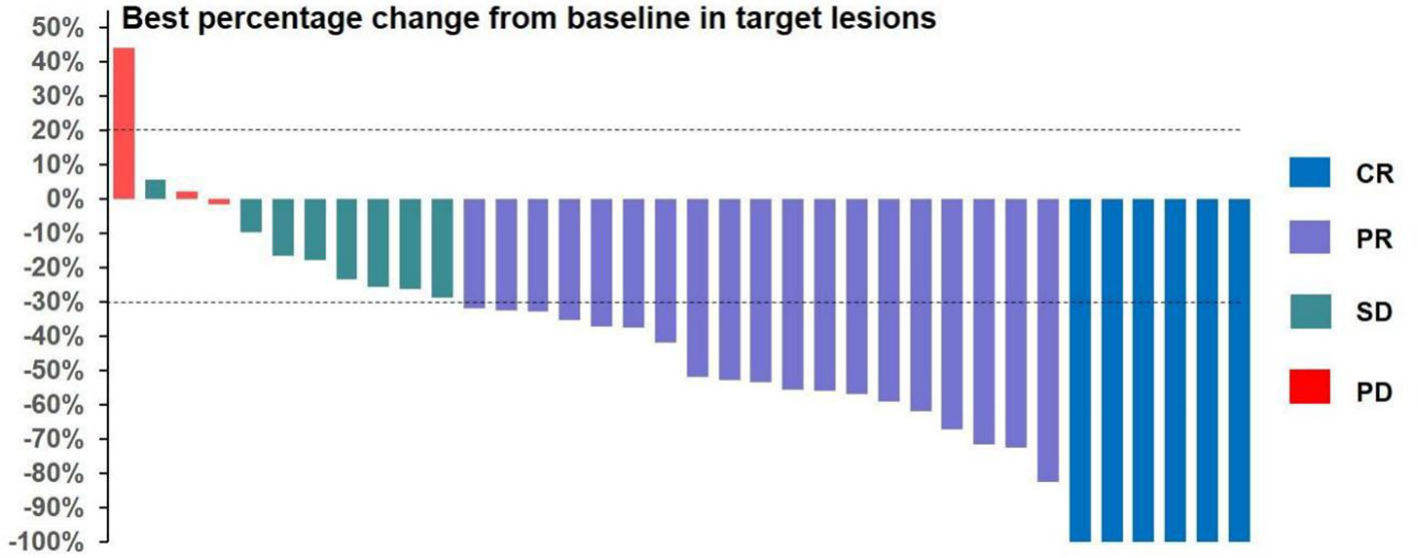
Bar chart showing best percentage change from baseline in target lesions.

All 36 subjects were eligible for efficacy evaluation, and the median number of TACE treatments was 2.3 (range: 1-5). According to the mRECIST, the ORR was 69.4% (25/36) and the DCR was 91.7% (33/36).Of the patients, 6 (16.7%) achieved CR; 19 (52.8%) achieved PR; 8 (22.2%) had SD; and 3 (8.8%) achieved PD (**Table 3**). Compared with baseline, the target lesion burden decreased in 32 (88.9%) patients. Six (16.7%) patients successfully underwent conversion surgery and achieved R0 resection. Two patients achieved complete pathological response. Notably, the frequency of liver function deterioration in patients with good tumor response (CR + PR) after treatment was 12.0%, which was significantly lower than that in patients with poor tumor response (SD + PD) at 27.3% (*P* < 0.05), suggesting that a change in the ALBI grade is a prognostic factor for tumor efficacy. ROC curve analysis revealed that the optimal cutoff value for ALBISC was −0.18, allowing for the classification of patients into two groups: a high-ALBISC group (ALBISC > −0.18) and a low-ALBISC group (ALBISC ≤ −0.18) (**Figure 3**). The ORR of the high-ALBISC group was 56.5%, whereas that of the low-ALBISC group was substantially higher at 84.6%. These findings indicate that ALBISC is a valuable prognostic biomarker, providing important insights for future treatment strategies. **Figure 4** presents the temporal changes from baseline in the target lesions (mRECIST).

**Table 3:** Tumor response according to mRECIST.

Variables	All patients (N = 36)
Best objective response, n (%)	
Complete response	6 (16.7%)
Partial response	19 (52.8%)
Stable disease	8 (22.2%)
Progressive disease	3 (8.8%)
Objective response rate, n (%), 95% CI	25 (69.4%) (51.9–83.7)
Disease control rate, n (%), 95% CI	33 (91.7%) (77.5–98.2)
TTR, months, range	1.4 (1.1–4.4)
DOR, months, median (95% CI)	11.4 (9.23–NA)
OS, months, median (95% CI)	NA (NA–NA)
PFS, months, median (95% CI)	10.7 (7.43–NA)

Unless indicated otherwise, values are presented as n (%).

CI, confidence interval; TTR, time to recurrence; DOR, duration of response; OS, overall survival; PFS, progression-free survival.

#### Survival analysis

**Figure 5:**
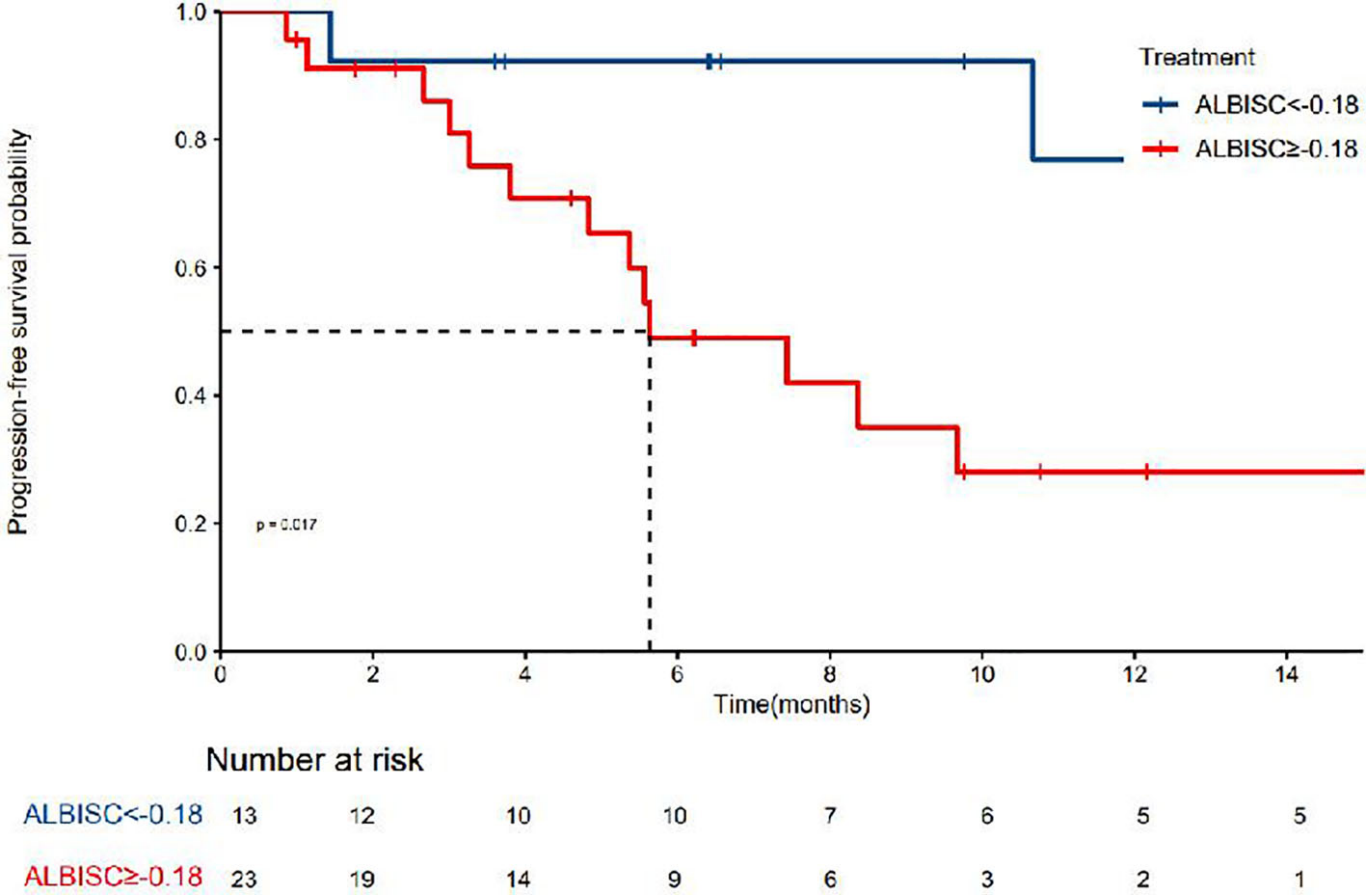
Kaplan-Meier survival plot showing progression-free survival probability over time.

**Figure 6:**
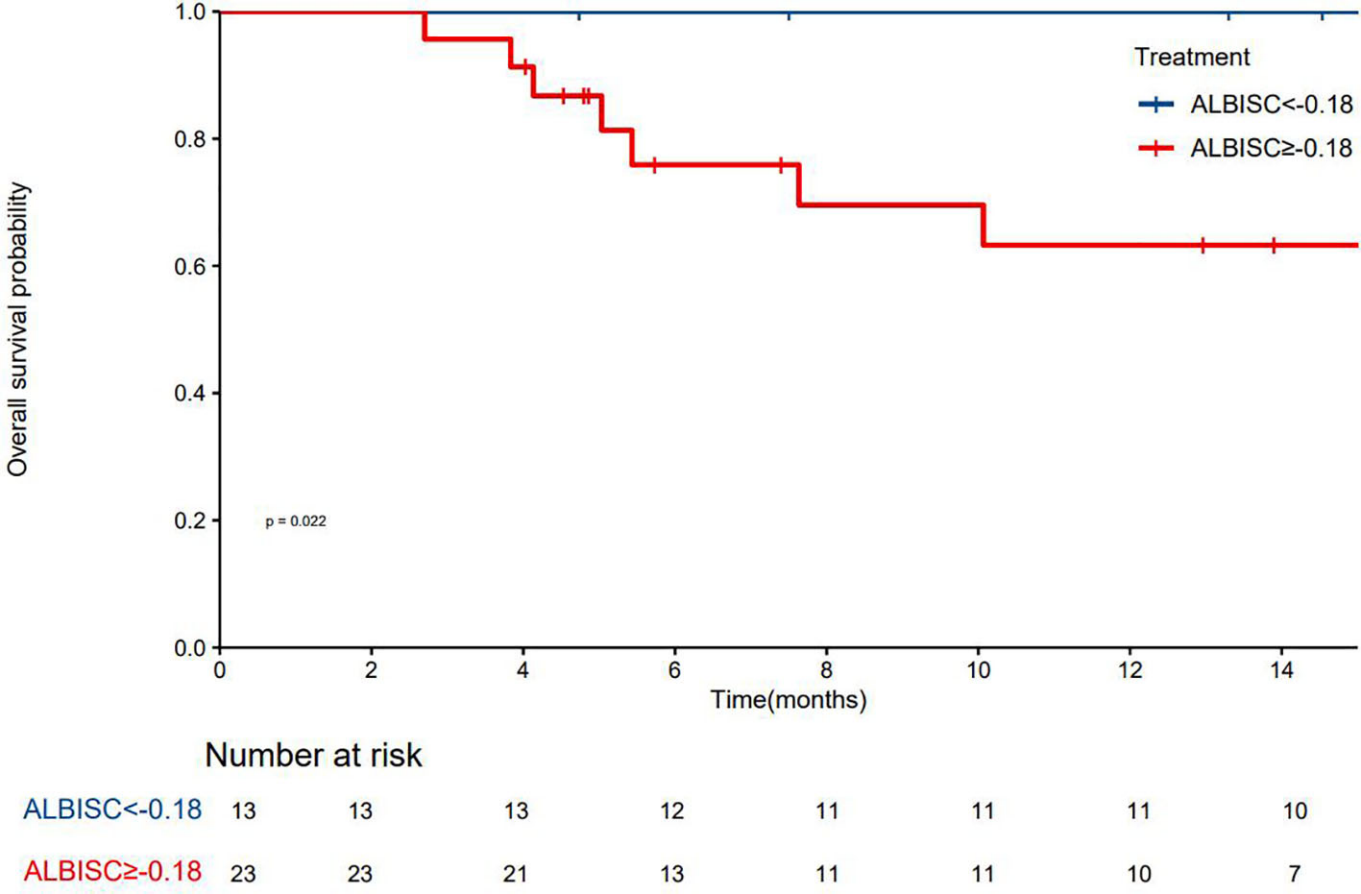
Kaplan-Meier survival curve showing overall survival probability over 14 months.

The median PFS was 10.7 months (95% confidence interval [CI]: 8.37–NA months, the median duration of response was 11.4 months, the median OS time was not reached, the median time to recurrence (mTTR) was 1.4 months (95% CI: 0.8–6.9 months), and the onset time was relatively fast(**Table 2**). For the Cox regression analysis adjusting for tumor stage and other baseline characteristics, we performed both univariate and multivariate analyses. In the univariate analysis, factors including ALBISC (HR: 2.34, 95% CI: 1.12-4.89, P=0.024), baseline ALBI grade, portal vein tumor thrombus, and alpha-fetoprotein levels were identified as potential prognostic factors. In the multivariate Cox regression model adjusted for BCLC stage, baseline ALBI grade, portal vein tumor thrombus status, and alpha-fetoprotein levels, ALBISC remained an independent predictor of progression-free survival (adjusted HR: 2.15, 95% CI: 1.03-4.47, P=0.041). This confirms that the prognostic value of ALBI grade change is independent of tumor stage and other baseline characteristics (**Table 4**). The ROC curve analysis revealed an optimal ALBISC cutoff value of −0.18, effectively distinguishing patients into low (−0.18 or lower) and high (>−0.18) ALBISC groups. The low-ALBISC group did not achieve PFS, whereas the high-ALBISC group had a median PFS of 5.63 months (*P* = 0.017) (**Figures 5**, **6**).

**Table 4:** Univariate and multivariate predictors of PFS.

Variable	Univariable analysis			Multivariable analysis		
	HR	95% CI	P value	HR	95% CI	P value
ALBISC (>-0.177 vs ≤-0.177)	2.34	1.12–4.89	0.024*	2.15	1.03-4.47	0.041
Sex (male vs. female)	0.89	0.34–2.31	0.812			
Portal vein tumor thrombus (Yes vs No)	1.89	1.02–3.51	0.043*	1.72	0.92-3.23	0.091
Baseline ALBI grade (2 vs 1)	1.78	0.94–3.37	0.076	1.34	0.69–2.61	0.388
AFP levels (≥400 vs <400 ng/mL)	1.67	0.91–3.07	0.098			
BCLC stage (C vs B)	1.56	0.84–2.89	0.158			
Immune checkpoint inhibitor (yes vs. no)	0.73	0.35–1.52	0.401			

*P < 0.05 indicates statistical significance.

HR, Hazard ratio; CI, confidence interval; ALBISC, albumin-bilirubin score change;AFP, alpha-fetoprotein;BCLC, Barcelona Clinic Liver Cancer;ALBISC was defined as the change in ALBI score from baseline to disease progression. The optimal cutoff value of -0.177 was determined by ROC curve analysis. Variables with P < 0.10 in univariate analysis were included in the multivariate model.

#### Adverse events

The combination therapy showed good tolerance, and no deaths from treatment-related AEs were reported. Throughout the treatment, 32 (58.2%) patients reported at least one AE. Among them, three experienced grade 3 AEs The primary AEs were elevated liver enzyme levels, thrombocytopenia, and fatigue. These findings highlight the importance of effectively controlling the tumor as well as carefully managing and monitoring patients’ liver health and other related factors.

## Discussion

Most patients with HCC are diagnosed at an advanced stage when surgical resection is no longer feasible, and the postoperative recurrence rate is high, resulting in poor overall prognosis (17). TACE is often recommended as the first-line treatment for advanced HCC in patients who are not candidates for surgical resection (18). However, the outcomes of TACE alone in the treatment of intermediate to advanced HCC are unsatisfactory. Targeted therapy and immunotherapy have provided more options for patients with unresectable and advanced liver cancer. In addition, multiple combination regimens are recommended as first-line treatment options. However, these therapies may lead to adverse reactions, such as liver function impairment, hypertension, and proteinuria (3, 19). Previous studies have demonstrated that the incidence of liver injury following molecular targeted drug therapy in patients with primary liver cancer ranges from 15% to 35% (20, 21), with common liver injuries being elevated transaminases and hyperbilirubinemia. The occurrence of severe AEs may lead to dose reduction, treatment discontinuation, or even treatment failure, which can affect antitumor efficacy and, in rare cases, result in patient death. Therefore, liver function plays a crucial role in combined targeted and immunotherapy against HCC.

In this study, donafenib + TACE therapy for patients with unresectable HCC yielded remarkable results. The ORR reached 69.4%, the DCR was as high as 91.7%, the mTTR was 1.4 months, the mPFS was 10.7 months, and the mOS had not yet been reached. These outcomes demonstrate superior efficacy compared to established standard treatments for intermediate and advanced HCC. Our combination therapy significantly outperformed TACE monotherapy in BCLC B patients, where ORR typically ranges from 15-25% and DCR from 60-70% according to recent meta-analyses (22, 23).Compared to systemic therapies for BCLC C patients, our results are also encouraging. Sorafenib monotherapy achieves ORR of approximately 2-3% with DCR of 43-54% (24), while the REFLECT trial showed lenvatinib achieving ORR of 18.8% and DCR of 75.3% (7).The recent IMbrave150 trial demonstrated that atezolizumab plus bevacizumab, representing the current standard of care, achieved ORR of 27.3% and DCR of 73.6% in advanced HCC (25). Our combination approach surpassed all these established therapies in terms of objective response rates.

Our median PFS of 10.7 months compares favorably with current standards. TACE monotherapy typically achieves median PFS of 4–6 months in BCLC B patients (26, 27).For BCLC C disease, sorafenib achieves median PFS of 4.1 months (24),lenvatinib 7.4 months (7),and atezolizumab plus bevacizumab 6.8 months (25).Notably, the LAUNCH trial, evaluating lenvatinib combined with TACE in advanced HCC, reported median PFS of 10.6 months (3), which is remarkably similar to our findings, suggesting comparable efficacy between these combination approaches.

The hepatoprotective profile of our combination represents a significant advancement over current treatments. Traditional TACE monotherapy is associated with liver function deterioration in 30-40% of patients, as noted in our study and confirmed by other reports (28). Systemic targeted therapies also carry substantial hepatotoxicity risks: sorafenib causes grade 3–4 liver dysfunction in 8-11% of patients (29),while lenvatinib shows similar rates of hepatic adverse events (30).In contrast, our study demonstrated remarkable liver function preservation, with 58.3% of patients maintaining stable hepatic function and 25% showing improvement from ALBI grade 2 to 1. Only 12.0% of responding patients experienced liver function deterioration, significantly lower than the 27.3% observed in non-responders. This hepatoprotective effect may be attributed to donafenib’s improved molecular stability and enhanced safety profile compared to sorafenib, as demonstrated in the ZGDH3 trial (lower incidence of drug-related adverse events: 85.3% *vs*. 95.5%) (12).Donafenib, as a new type of multitarget TKI, exerts inhibitory effects on the VEGF and PDGF pathways, which can block tumor angiogenesis and growth and improve the embolization effect of TACE (31). Meanwhile, local TACE treatment can induce tumor cell necrosis and activate the body’s antitumor immune response, synergizing with donafenib in the antitumor effect (32). The unique molecular structure and pharmacokinetic properties of donafenib further reduce adverse reactions and enhance safety (10), explaining the synergistic antitumor and hepatoprotective effects observed in our study.

The combination of high response rates with liver function preservation addresses a critical gap in HCC management. Unlike conventional approaches that often compromise hepatic safety for therapeutic efficacy, our regimen achieves both objectives simultaneously, establishing a new treatment paradigm for BCLC B and C patients.

The 16.7% conversion surgery rate is particularly meaningful given the advanced disease characteristics of our cohort, including portal vein tumor thrombus (30.6%) and elevated alpha-fetoprotein ≥400 ng/mL (50.0%). Successful conversion to resectable disease provides long-term survival opportunities unattainable with palliative therapies alone. The rapid treatment response (mTTR 1.4 months) offers additional advantages for patients requiring urgent tumor control.

In this study, ROC curve analysis revealed that changes in the ALBI grade could serve as a predictor of therapeutic efficacy, with patients achieving ALBISC ≤−0.18 showing significantly superior ORR, PFS, and OS compared to those with ALBISC >−0.18. Unlike previous studies (33, 34) that focused on baseline ALBI grades for prognostic assessment, our findings establish dynamic ALBI grade changes as treatment response predictors, providing clinicians with a practical tool for therapy optimization.

Safety dimension analysis revealed that the incidence of adverse reactions was 58.2%, only three patients experienced grade 3 adverse reactions, and no treatment-related deaths occurred, reflecting the safety and tolerability of donafenib, consistent with the findings of other domestic studies (35).This improvement in safety may result from its modified molecular structure reducing the production of toxic metabolites. However, the safety margin in specific patient groups, such as Child–Pugh grade B, still needs to be carefully evaluated.

This single-arm study design limits direct comparisons with standard treatments. Randomized controlled trials are needed to provide definitive comparative evidence. Longer follow-up will establish overall survival benefits and assess the durability of hepatic function preservation. The safety profile in Child-Pugh grade B patients requires evaluation in larger cohorts.

## Conclusion

In general, the combination of TACE and donafenib, with or without PD-1 monoclonal antibody, for the treatment of advanced HCC is safe. This combination therapy can enhance the therapeutic effect and liver function of unresectable HCC. Thus, it can be a novel treatment strategy for advanced HCC.

## Data Availability

The datasets presented in this study can be found in online repositories. The names of the repository/repositories and accession number(s) can be found in the article/supplementary material.
